# Renal cell carcinoma associated with peritumoral sarcoid-like reaction without intratumoral granuloma

**DOI:** 10.1186/1746-1596-7-28

**Published:** 2012-03-18

**Authors:** Simon Ouellet, Roula Albadine, Robert Sabbagh

**Affiliations:** 1 Department of Surgery, Division of Urology, Sherbrooke University, Centre hospitalier universitaire de Sherbrooke, 3001, 12e Avenue Nord, Sherbrooke, QC J1H 5N4, Canada; 2 Department of Pathology, Sherbrooke University, Centre hospitalier universitaire de Sherbrooke, 3001, 12e Avenue Nord, Sherbrooke, QC J1H 5N4, Canada

**Keywords:** Sarcoid-like reaction, Granuloma, Renal cell carcinoma

## Abstract

Non-necrotizing epithelioid granulomas have been described in association with many primary tumors. In such cases, they are designated as sarcoid-like reaction. Although it is more seen in carcinomas than in sarcomas, it is very rarely reported in renal carcinoma. Here, we describe a rare association of prominent peritumoral sarcoid-like reaction without intratumoral granulomas and conventional clear cell renal carcinoma in a 62-year-old-male, without clinical or laboratory finding of sarcoidosis. At 30 months follow-up, he had no recurrence.

**Virtual Slides:**

The virtual slide(s) for this article can be found here: http://www.diagnosticpathology.diagnomx.eu/vs/4054525336657922

## Background

Non-necrotizing granulomas have been described in association with several primary cancers. Histologically, this sarcoid-like reaction is undistinguishable from granulomas found in systemic sarcoidosis. It is composed of a focal accumulation of epitheloid cells and multinucleated giant cells [1]. Few cases of association of sarcoid-like reaction with renal cell carcinoma have been described [2-6], and some in patients with a known or suspected systemic sarcoidosis [7-10]. Here, we describe a renal cell carcinoma associated with a peritumoral granulomatous reaction in a patient without systemic sarcoidosis.

## Case presentation

### Case report

**Figure 1:**
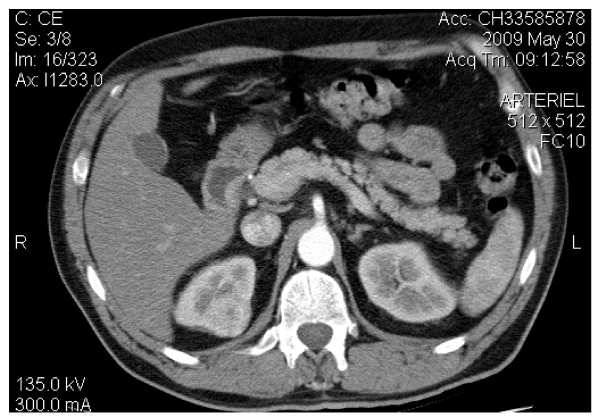
**CT of the abdomen**. Heterogeneous enhancing lesion in the upper pole of the right kidney without invasion to surrounding tissues.

A 62-year-old caucasian male known for dyslipidemia and scalp psoriasis was admitted to the emergency room for right renal colic. The patients had no history of constitutional symptoms, gross hematuria or abdominal pain. Laboratory findings were unremarkable. A computerized tomography (CT) was performed, which showed a 3.3 cm heterogeneous enhancing lesion in the upper pole of the right kidney consistent with a renal carcinoma (Figure 1). Patient was then scheduled for a laparoscopic partial nephrectomy. The per- and post-operative periods were uneventful. Lymph nodes were explored during surgery and none were found. Nothing in the patient's clinical history or in the thoracic and abdominal CT scan performed suggested sarcoid granulomas involvement. No lymph node nor metastasis were present at the time of the surgery and at 30 months follow-up.

### Pathologic findings

Macroscopically, the tumor lesion revealed a 3.5 cm encapsulated yellowish mass with bosselated surface with small foci of hemorrhage and necrosis.

**Figure 2:**
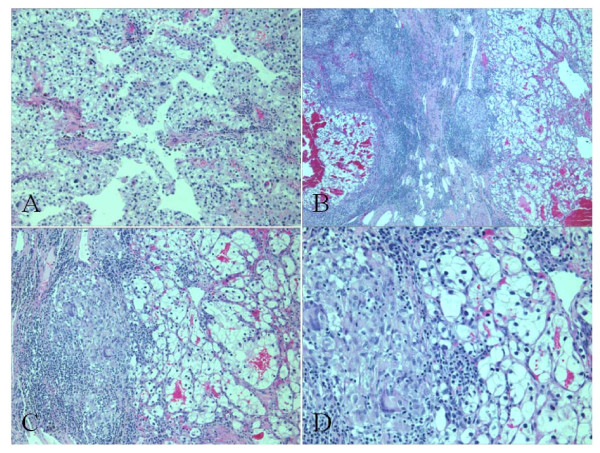
**Partial right upper pole nephrectomy**. A, Conventional clear cell type renal carcinoma of Fuhrman nuclear grade III, without sarcomatoid features (HE;×100 original magnification). B, Epithelioid cell granulomas with Langhans-type giant cells (Sarcoid-like reaction) in the peritumoral fibrous pseudocapsule (×40 original magnification). C, Sarcoid-like reaction with some peritumoral inflammatory reaction, without any contact with tumor cells (×100 original magnification). D, Renal cell carcinoma with sarcoid-like reaction (×200 original magnification)

**Figure 3:**
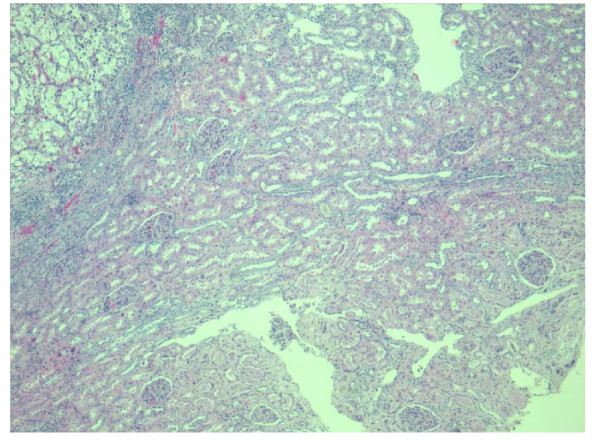
**No granuloma was observed in the adjacent normal renal parenchyma (x40 original magnification)**.

Histological examination showed a conventional clear cell type renal carcinoma of Fuhrman nuclear grade III, without sarcomatoid features (Figure 2A). There was no perinephric, renal sinus fat, or renal vessel involvement. Surgical margins were negative. Neoplastic proliferation was delineated from normal renal parenchyma by a fibrous pseudocapsule where multiple non-necrotizing granulomas with multinucleated giant cells were found (Figure 2B, C, D). No granuloma was seen within the tumor. These granulomas did not contain centrally located malignant cells. These granulomas were associated with mild mononuclear, lymphocytic inflammatory infiltrate. No granuloma was seen in the adjacent renal parenchyma (Figure 3). Ziehl-Neelsen and Grocott stains did not detect the presence of mycobacteria or fungi.

## Discussion

The frequency of sarcoid like reactions in certain tumor types and in different locations varies from 4% in carcinoma, to 20% in lymphoma [1]. Non-caseating granulomas can be caused by chemical exposure, infections, foreign bodies, granulomatous diseases and tumors [1,11]. Therefore, before claiming an association between renal cell cancer and sarcoid-like reaction, the other causes must be excluded by a careful clinical history, diagnostic tests and pathologic examination [5].

Antigens expressed by the neoplastic cell or soluble tumor antigens trigger an immune response which leads to the formation of non-caseating granulomas. Such reaction, locally mediated by T-cell, can be found in involved or uninvolved remote site, in regional lymph nodes and less frequently in tumoral areas [1-11].

A certain number of cases in the literature reports sarcoid-like reaction associated with renal cell carcinoma in patient with sarcoidosis [7-10]. Renal involvement in sarcoidosis displays a wide range of clinical manifestations. Renal histopathology shows granulomatous interstitial nephritis. Alexandrescu et al [12] reported one case of renal cancer with non-cutaneous sarcoidosis. Lucci et al [10] described the 6^th ^case of clear cell renal cell carcinoma associated with sarcoidosis, this association is very rare.

Few cases reported sarcoid-like reaction associated with renal cell carcinoma in patients without sarcoidoisis [2-6]. We presented the sixth case of this reaction in conventional clear cell type renal carcinoma, in patients without sarcoidosis (Table 1). The prognostic factor of this association is still unclear. Kamiyoshihara et al [13] found no difference regarding lung cancer prognosis. However, Pavic et al [14] suggested that sarcoid-like reaction could play a role in preventing metastatic dissemination and may be associated with a better prognosis in Hodgkin's disease and gastric adenocarcinomas. Piscioli et al [6] reported a case of a renal cell carcinoma with sarcomatoid and sarcoid-like reaction. The patient died from metastatic dissemination 6 months after the nephrectomy. The authors suggested that his poor prognostic was not influenced by the sarcoid-like reaction but rather the sarcomatoid features. However two other cases reported longer recurrence free survival of 15 [4] and 48 [3] months, respectively. In our case, the sarcoid like reaction was seen peritumoral, without sarcomatoid features, with a recurrence free survival at 30 months follow-up. Sarcoidosis and sarcoid-like granulomas have been associated with psoriasis treatments [15,16]. However, in our case the patient never had any treatment for his scalp psoriasis.

**Table 1:** Clinicopathological features of reported cases of sarcoid-like reaction associated with conventional clear cell type renal carcinoma in patients without sarcoidosis

Reference	Age (y)/Sex	Histology	Tumor size (cm)	Follow-up
Hes et al. [3] (2003)	73 to 85 (mean 78.3)/2M, 1F	Granulomatous reaction within tumorous stroma	2.3 to 7.0 (mean 4.4)	6 months to 4 years

Kovacs et al. [4] (2004)	62/F	Granulomatous reaction within tumorous stroma and fibrous stroma surrounding the tumor	6.0	15 months

Shah et al. [5] (2010)	62/M	Granulomatous reaction within tumorous stroma	5.0	12 months

Present case	62/M	Peritumoral sarcoid-like reaction without intratumoral granulomas	3.5	30 months

Interestingly, in our case we did not find granulomatous reaction within the tumor as described in some carcinoma, including breast [17], renal [2-5] and hepatocellular carcinomas [18]. We presented the sixth case of this reaction in conventional clear cell type renal carcinoma, in patients without sarcoidosis. However, it is, to our knowledge the first case of conventional clear cell type renal carcinoma to show peritumoral sarcoid-like reaction without intratumoral involvement.

## Conclusion

We report a rare association between conventional clear cell type renal carcinoma and peritumoral sarcoid-like granulomatous reaction in a patient without clinical, radiologic or laboratory finding of sarcoidosis. Due to the low number of published cases, prognostic value of peritumoral non-necrotizing epithelioid granulomas has yet to be determined. Further cases are needed to provide information on the mechanism and prognostic value of peritumoral granuloma reaction in renal cell carcinoma.

## Consent

Written informed consent was obtained from the patient for publication of this Case Report and any accompanying images. A copy of written consent is available for review by the Editor-in-Chief of this journal.

## Competing interests

The authors declare that they have no competing interests.

## Authors' contributions

SO drafted the manuscript and provided clinical information. RA carried out the histological evaluation and helped drafted this manuscript. RS was the surgeon, supervised and helped drafted this manuscript. All authors read and approved the final manuscript.
